# Supplementary material for: Bioengineering secreted proteases converts divergent Rcr3 orthologs and paralogs into extracellular immune co-receptors
Source: Plant Cell. 2024 Jun 26;36(9):3260–76. doi: 10.1093/plcell/koae183 (PMC11371160; doi:10.1093/plcell/koae183)
Supplement: koae183_Supplementary_Data [file koae183_supplementary_data.zip › Supplementary Figures.pdf]

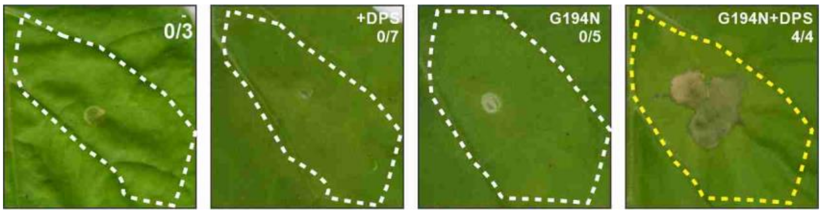

Supplementary Figure S1 r*Nb*Rcr3(G184N+DPS) induces Avr2/Cf-2-dependent HR. (Supports **Figure 2**)
Resurrected (r) *Nb*Rcr3 and derived +DPS and G194N mutants were co-expressed with Avr2 and Cf-2 by agroinfiltration of *Nicotiana benthamiana* with OD = 0.25 each in a 1:1:1 ratio. Images were taken at 5dpi. Numbers indicate the number of agroinfiltrated sectors showing HR symptoms. Dashed lines highlight sectors without HR (white) or with HR (yellow).

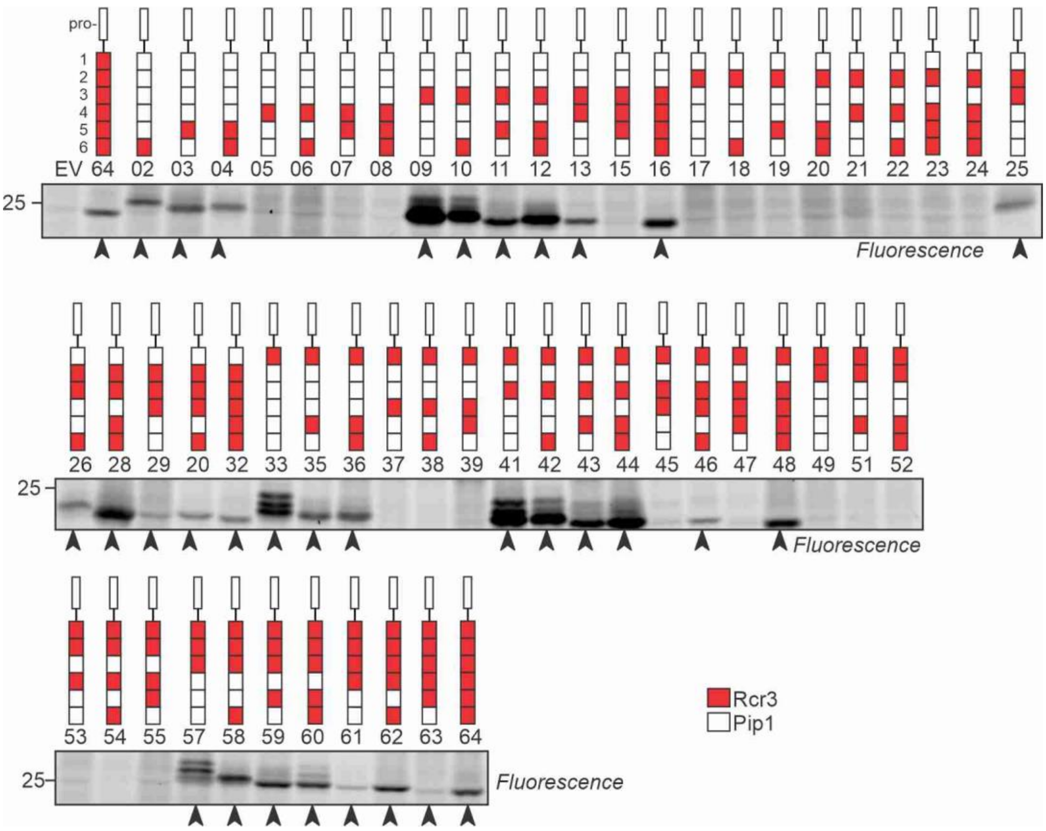

Supplementary Figure S2 Thirty-three Pip1/Rcr3 hybrids are active proteases. (Supports **Figure 3**)

Apoplastic fluids isolated from agroinfiltrated leaves transiently expressing Rcr3/Pip1 hybrids at 5 days post-infiltration (5 dpi) were labelled for 5 hours with 2μM MV201. Samples were separated on SDS-PAGE gels and scanned for fluorescence. Active hybrid proteases are indicated with arrowheads.

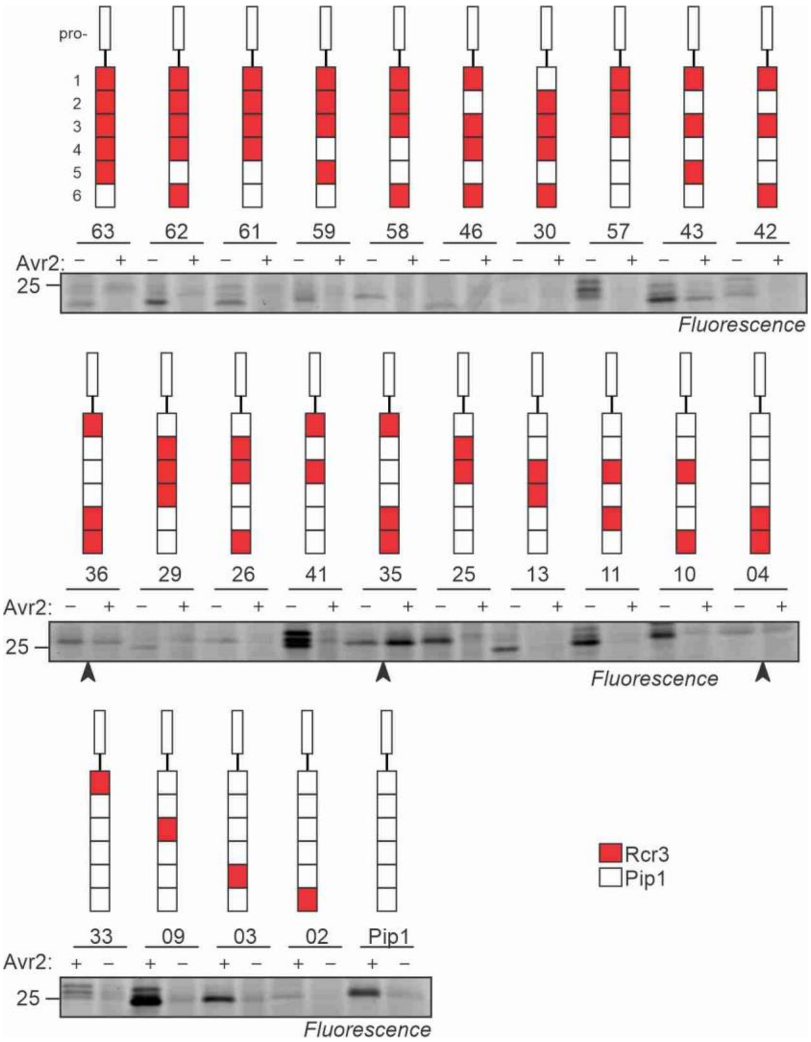

Supplementary Figure S3 Thirty Pip1/Rcr3 hybrids can be inhibited by Avr2. (Supports **Figure 3**) Apoplastic fluids isolated from agroinfiltrated leaves transiently expressing Rcr3/ Pip1 hybrids at 5 days-post-infiltration (5dpi) were pre-incubated for 45min with and without 500 nM Avr2 and labelled for 5 hours with 2μM MV201. Samples were separated on SDS-PAGE gels and scanned for fluorescence. The three hybrid proteases that cannot be inhibited by Avr2 are indicated with arrowheads.

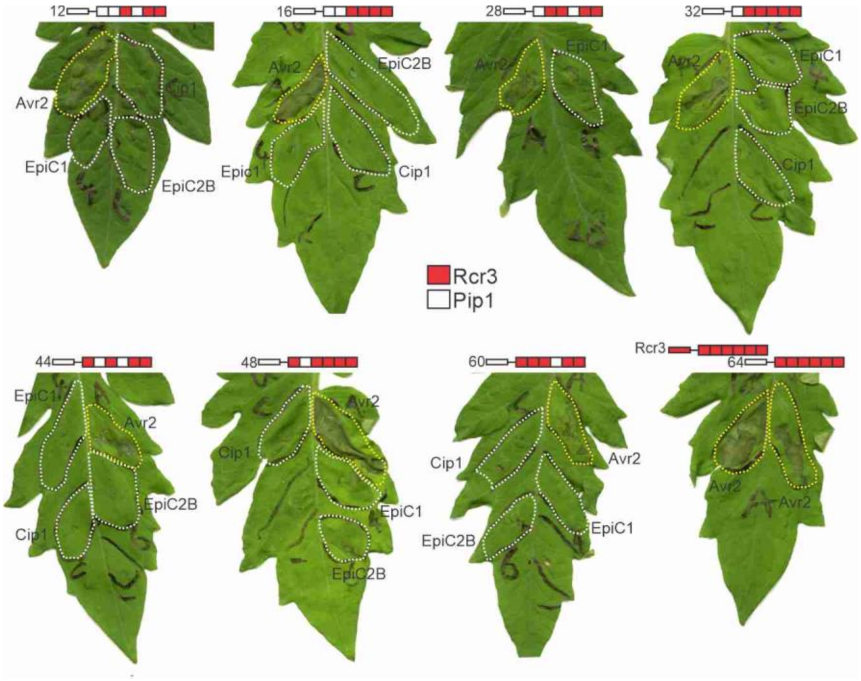

Supplementary Figure S4 Eight Pip1/Rcr3 hybrids trigger Avr2/Cf-2-dependent HR. (Supports **Figure 3**). Apoplastic fluids isolated from agroinfiltrated leaves transiently expressing Rcr3/Pip1 hybrids at 5 days post-infiltration (5 dpi) were co-infiltrated with 1µM purified recombinant Avr2, Cip1, EpiC1 or EpiC2B into leaflets of tomato MM-*Cf-2 rcr3-3* plants. Only co-injection with Avr2 triggers HR (yellow lines). Pictures were taken at 5 dpi. Dashed lines highlight sectors without HR (white) or with HR (yellow).

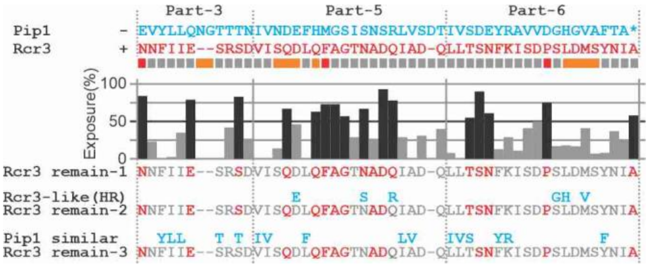

Supplementary Figure S5 Selection of Rcr3-specific residues that may contribute to HR. (Supports **Figure 4**). Variant residues in parts -3, -5 and -6 are summarized and the outcome of the quantitative HR assay is summarized are shown below with residues that are irrelevant (grey), important (red) or collectively required (orange) for HR. The exposure of each residue in the structural model of Rcr3 was calculated using the 'get_sasa_relative' command in PyMol, and excluded if below 50% (remain-1). Variant residues present in Rcr3 orthologs from *Solanum* species that are identical to the residue detected in Pip1 are also excluded (remain-2). Residues that are chemically very similar between Rcr3 and Pip1 are also excluded (remain-3).
