# Supplementary material for: Bioengineering secreted proteases converts divergent Rcr3 orthologs and paralogs into extracellular immune co-receptors
Source: Plant Cell. 2024 Jun 26;36(9):3260–76. doi: 10.1093/plcell/koae183 (PMC11371160; doi:10.1093/plcell/koae183)

Figure 1E)

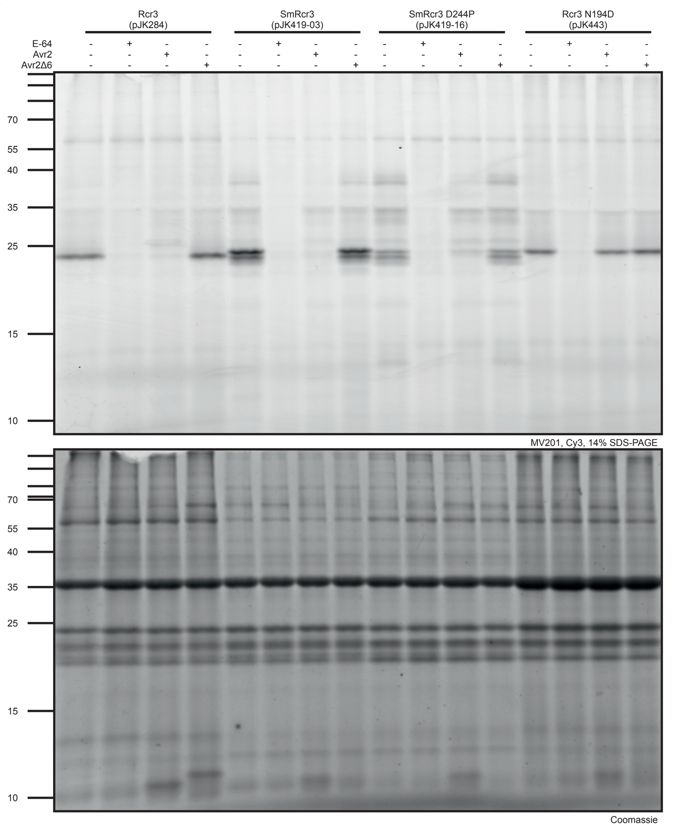

Figure 2B)

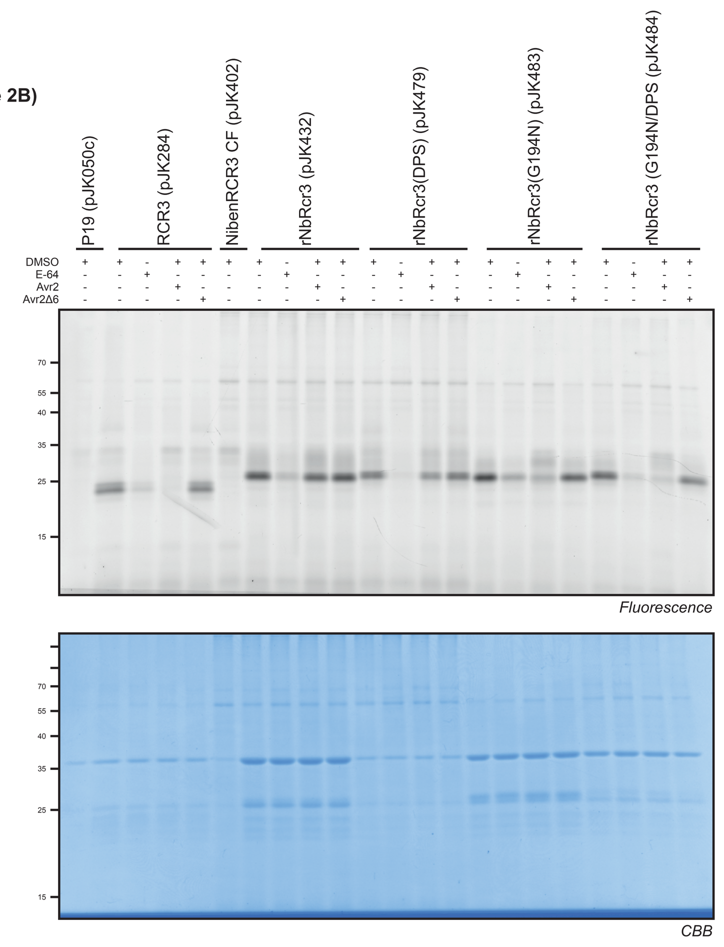

Figure 2C)

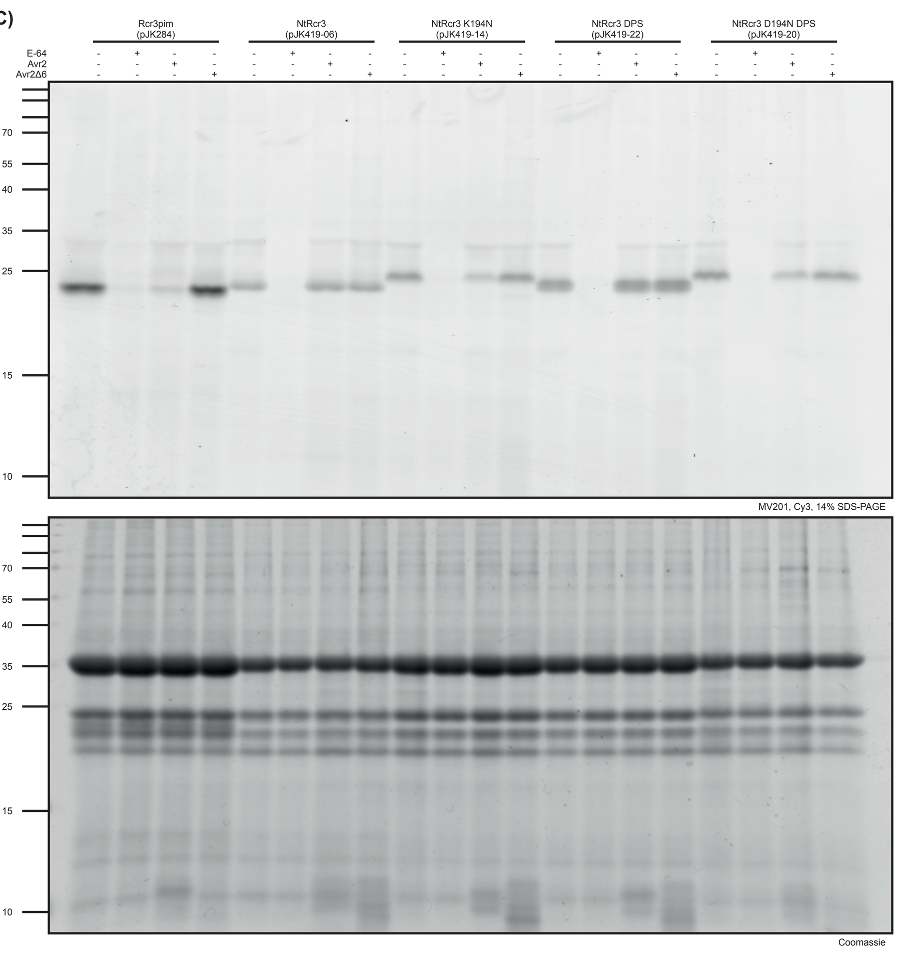

Figure 3C)

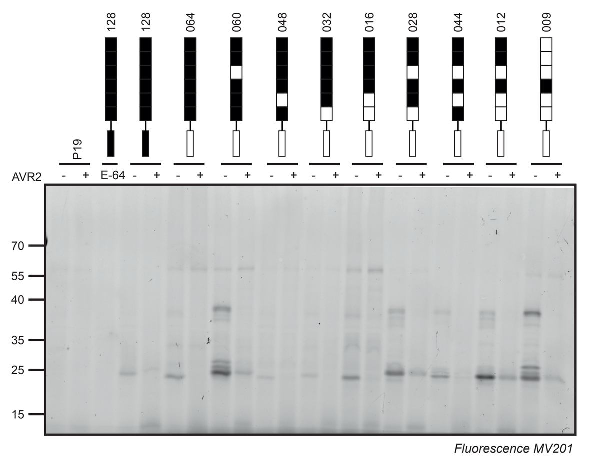

Figure S1)

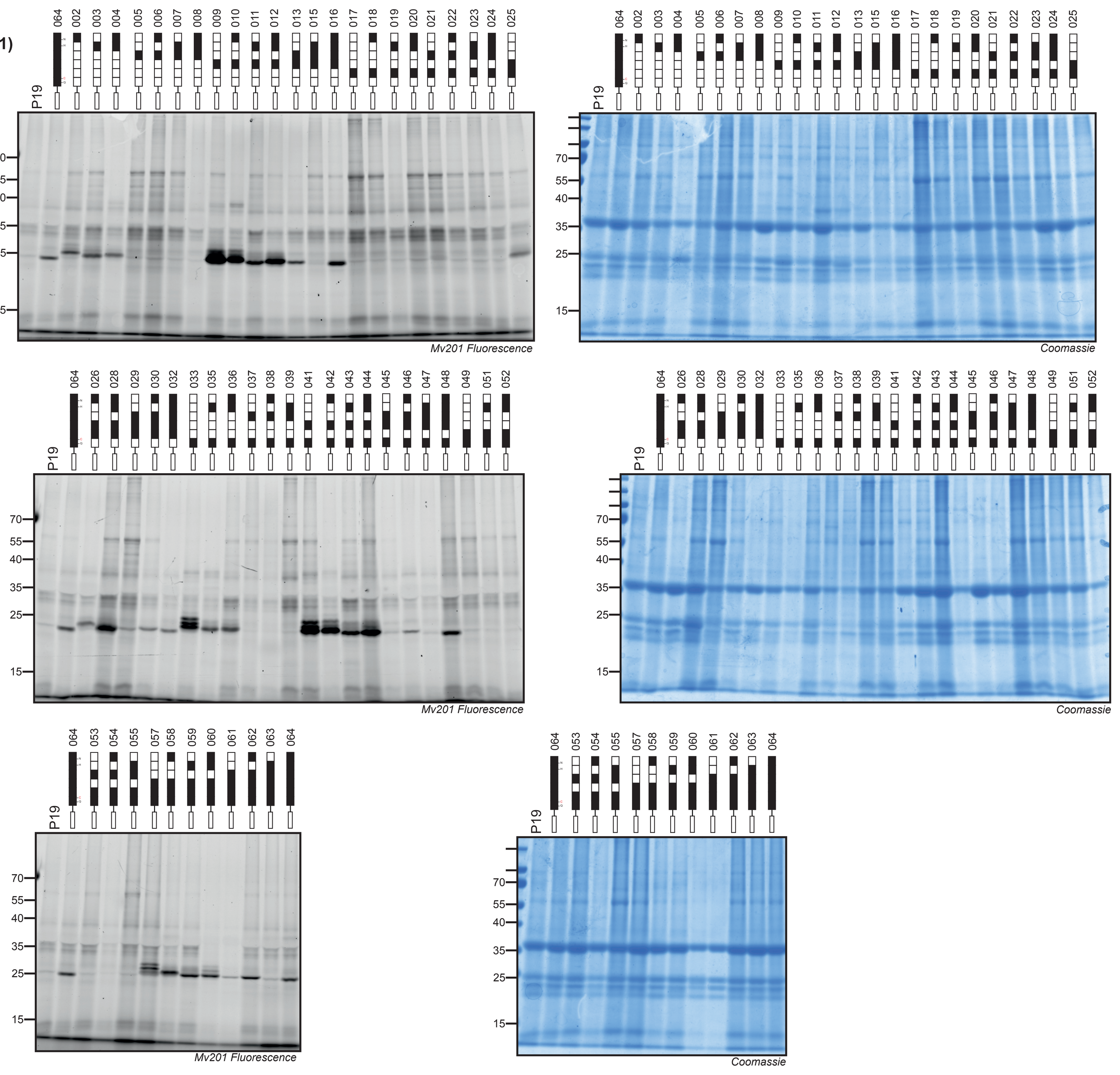

Figure S2)

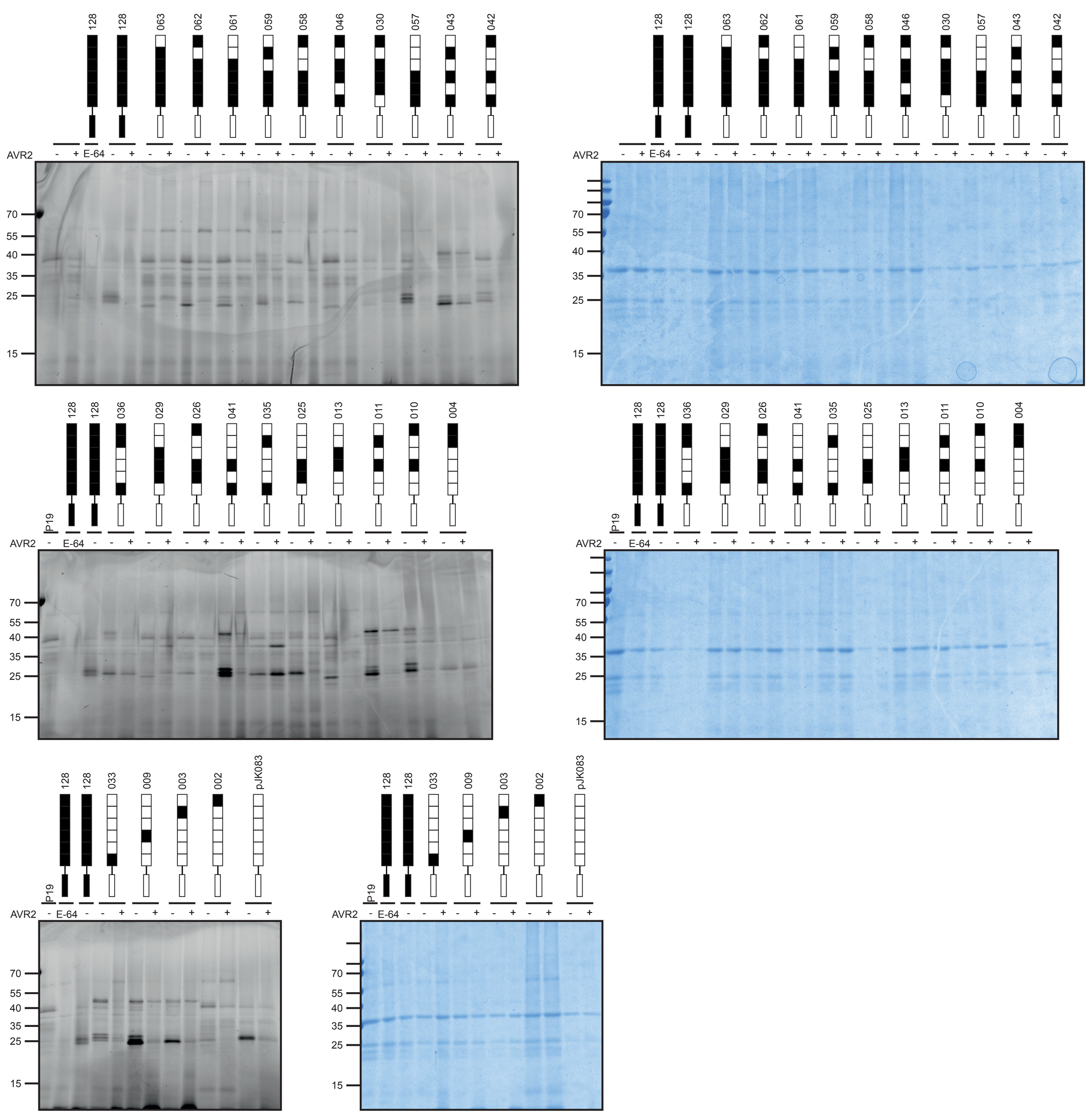

Supplement: koae183_Supplementary_Data [file Supplementary Dataset S1.pdf › Supplementary Dataset S1.pdf]
