# Supplementary material for: Bioengineering secreted proteases converts divergent Rcr3 orthologs and paralogs into extracellular immune co-receptors
Source: Plant Cell. 2024 Jun 26;36(9):3260–76. doi: 10.1093/plcell/koae183 (PMC11371160; doi:10.1093/plcell/koae183)
Supplement: koae183_Supplementary_Data [file tpc.00175.2024-s09.pdf › tpc.00175.2024-s09.pdf]

THE PLANT CELL

Bioengineering secreted proteases converts divergent Rcr3 orthologs and paralogs into extracellular immune co-receptors

Jiorgos Kourelis, Mariana Schuster, Fatih Demir, Oliver Mattinson, Sonja Krauter, Parvinder Kahlon, Ruby O'Grady, Samantha Royston, Anna Lucia Bravo-Cazar, Brian Mooney, Pitter Huesgen, Sophien Kamoun, and Renier van der Hoorn

Review Timeline:

Submission Date:	22-Feb-2024
Editorial Decision:	06-Apr-2024
Revision Received:	24-May-2024
Accepted:	28-May-2024

Dear Dr. van der Hoorn:

We have received reviews of your manuscript entitled "Bioengineering secreted proteases converts divergent Rcr3 orthologs and paralogs into extracellular immune co-receptors." On the basis of the advice received, the board of reviewing editors would like to accept your manuscript for publication in The Plant Cell. This acceptance is contingent on revision based on the comments of our reviewers. In particular, please consider the following:

1) Both reviewers noted issues with how HR scores are represented throughout this manuscript. We concur that the present graphs are difficult to interpret and should be modified. In particular, it is not clear whether each construct is scored with the same number of leaves. If not, the use of bubble sizes to indicate number of injections with each score may be misleading. It is also unclear why some dots are inside the bubbles and some dots are outside the bubblles. Perhaps it would be easier to interpret with a box and whisker style graph? At a minimum, please add a value for the mean HR score and the total number of leaves injected for each construct (if the number varies from construct to construct).

2) The primary novelty of this work is the potential to engineer new disease resistance traits using an apoplastic decoy. However, this particular example seems fairly limited to detection of protease inhibitors. Please expand your discussion of the potential applications of this specific system and its limitations (e.g., can Rcr3-Cf2 be engineered to recognize proteins secreted by other fungal species?).

3) The protease inhibition assay employed in Figure 2 needs to be explained in the legend and in the text.

4) Both reviewers have provided detailed suggestions for improving clarity of the writing. Please take these into consideration as you revise your manuscript.

Please highlight all changes and include a detailed annotation of changes of the text, with line numbers, and noting your responses to the comments.

To submit your revised manuscript, click:

Link Not Available

If you have any questions about the revision submission procedures, please contact the Editorial Office Staff (tpc-submissions@aspb.org). If you cannot return the revised manuscript within 30 days, please let us know. Otherwise, we will assume that you have elected not to revise the manuscript and withdraw it.

Thank you very much for the privilege of reviewing this work. I look forward to receiving the next version.

Sincerely,

The Plant Cell Editorial Board

(Please note that due to a potential conflict of interest, EIC Blake Meyers did not participate in evaluating this manuscript).

Reviewer #1 (Comments for the Author):

The manuscript by Kourelis and colleagues titled "Bioengineering secreted proteases converts divergent Rcr3 orthologs and paralogs into extracellular immune co-receptors" tackles engineering proteases involved in plant immunity. The authors use knowledge gained from Proteins in one species /cultivar to swap amino acid residues and engineer increased recognition and resistance in the form of a hypersensitive response. This type of incremental work and tinkering are essential in transferring information gathered from one study and applying it to other proteins, which would help the community in identifying common mechanistic insights. I applaud the authors on the amount of work put in to generate and test these variants. The authors prove that by replacing specific amino acid residues proteins, in this case Rcr3 and Pip1 can be engineered in different species, which was one of the primary goals of the study.

I am also not convinced with the claim in the abstract that this study advances our understanding of the evolution of Avr2 perception. I agree that the engineering part of the manuscript is solid and the authors should stick to insights gained from this rather than make claims about evolution that dont stand.
The manuscript is largely well put together, but could do with some editing for clarity, especially given that the manuscript switches between different proteins in different species. It becomes difficult to follow the nomenclature and all the different amino acid variants. Please disregard my queries asking for the basis of classifying the Rcr3 protein into different parts, I see that its mentioned in the methods section that these are arbitrary. But this in a sense underscores my point about manuscript clarity. The figures supplied would also benefit from better illustration and organization. I specify points below aimed at improving the manuscript in its current form.

Major points:
1. Please rephrase "A single residue substitution in eggplant SmRcr3 is required for Avr2/Cf-2-dependent HR" to "A single residue substitution in eggplant SmRcr3 is sufficient for Avr2/Cf-2-dependent HR"
2. It is not clear in the manuscript the classification based on which the Rcr3 protein was split into parts. This information would be helpful in understanding why replacing certain parts lead to cf-2 signalling.
3. Figure. 1A - could you provide a better quality figure and if possible consider a different colour scheme, so that the figure is easier to read.
4. Figure. 1C - the HR indexes are difficult to read. This is true for all figures with HR indices.
5. Line 121 - "Most other variant residues in part-4 are also different between Rcr3 homologs that can trigger Cf-2/Avr2-dependent HR." I am confused with this point, arent the variants based on the difference between HR-triggering Rcr3 vs SmRcr3?
6. Lines 72-76 in the introduction suggest that the protease activity of Rcr3 is not required for Cf-2-dependent HR, so I find line 131 a bit misleading in claiming that the D244P substitution turns SmRcr3 into a protease that triggers Cf-2-dependent HR. Perhaps, it is more accurate to state that SmRcr3 with the D244P substitution is capable of triggering Cf-2-dependent HR? Please clarify and elaborate in the text, is the above is incorrect.
7. The image presented in Fig. 1D and the HR index suggest that the D244P substitution while displaying HR in SmRcr3 is not comparable to Rcr3. Am I correct in interpreting this? And could the authors elaborate on this either way?
8. Line 138 - I am not sure 'resurrected' is the best word to describe Rcr3a, but this is a semantic choice and I leave it to the authors to change or retain this. I would strongly suggest that they find a more suitable word.
9. Suggestion - G194N instead of N194 in the title (line 134) would be more informative.
10. The basis of Fig. 2 is mainly dependent on "DPS" insertion and a Asparagine (N) at position #194. Substitution of the above residues in NtRcr3 is clear (Fig. 2c), however I am not convinced with the data presented for "rNbRcr3" (Fig.2b). In the absence of deletion of these three residues in Rcr3, it is difficult to interpret the importance of these residues. That said, I think that deletion of these residues in Rcr3 will be tangential to his work. So I would ask the authors to provide a better image and if it helps add a supplementary data figure with just three comparisons Rcr3 vs rNbRcr3 vs G194N+DPS.
11. Line 166 - It is not clear what D329H refers to? Is it D329 in SlPip1 which naturally occurs at H329 in SpPip1? I strongly encourage the authors to go through all such attributions, and ensure that these are easier to read.
12. See point 2, what was the basis of splitting Rcr3 into seven "distinct" fragments?
13. Lines 292-293 need to be qualified as a possibility, as they are rightly done in the preceding lines.
14. Considering that Fig. 6c is based on Alphafold2, have the Authors explored the possibility of alternate structural arrangements of M338 and I285? Would a different arrangement open the substrate-binding pocket to accommodate other substrates?
15. Line 298-299 - "Thus, the larger S2 pocket in Pip1 explains why it can accommodate substrates with P2=Phe, in contrast to Rcr3, which prefers smaller residues at P2." - This conclusion is based on a premise built over an unverified model. The authors would be better off toning down these claims.

Minor points:
1. Specify tomato Rcr3 in line 37. I understand that this is a continuation of the previous line where Rcr3 in tomato was mentioned, however, I had to re-read this line a couple of times and think that simply assigning it as SlRcr3 or something equivalent for the introgressed line would make it easier to read.
2. Line 72 - Please expand on "not processed by Rcr3", I am assuming this refers to RCr3's protease activity.
3. Line 73-76 - please consider splitting this into two sentences.
4. Line 178 - cause → caused
5. Legend of Fig. 5a is incomplete
6. Line 287 - "also" is misplaced.
7. Line 331 - misspelled
8. Line 398 - please rephrase in the first line of the sentence, interaction between cf-2 and what? I assume authors mean Avr3 and Rcr3, which come at the very end of the sentence.
9. Line 409 - "Nevertheless" can be deleted

Reviewer #2 (Comments for the Author):

The manuscript describes the bioengineering of the plant-secreted immune proteases Rcr3 and Pip1 for gain of function in Cf-2 mediated recognition. The manuscript is well-written and should be of interest to the scientific community given the recent intense interest in receptor engineering. However, the findings would be of more interest by combining some selection analyses including newly available genomes.

Major comments:
The authors discussed different selection pressures may be present in a region and lineage specific bases. Specifically, HR-related Rcr3 residues may be under purifying or neutral selection. The paper would be strengthened by directly testing this,

potentially with codon-substitution models from CodeML.
When analyzing Rcr3 sequences in eggplant and progenitor species - are there any variants with D224P?

HR index: An average HR index should also be included in the figures, which will allow for a more straightforward comparison of HR intensity between constructs. Fig 3D: I suggest highlighting the HR inducing hybrid names with underscore or color for a more consistent and easier visual interpretation. For the raw dots in the bubble plot, it seems that not all hybrids have the 12 datapoints (are these replicates described in the legend?) plotted in the bubbles (E.g. hybrid 28, 32, 44, 48, 60 only have 7, 7, 6, 5, 7 data points, respectively). The uneven number of plotted data points may affect the size of bubbles as the bubbles seems to be direct representation of number of plotted data points.

AlphaFold Modeling: Please put Fig 7 data earlier to highlight in each fig for GOF regions that are required for recognition. Does a structure-based alignment of the native Rcr3 homologs including Pip1, Rcr3 from various species and bioengineered Rcr3 variants in this study reveal that HR-inducing homologs structures form a distinct cluster from non-HR inducing homologs? Provide pTM scores as quality control for the AlphaFold models in the supplemental material.

Protein expression: It would be good to demonstrate for a few key GOF mutants and negative controls that the PLCP is expressed by western blot.

Other comments:

Fig 3C: It seems that this fluorescence panel does not contain native Pip and Rcr3 as controls, and hybrid 48, 32, 128 seems to have very weak change of Fluorescence. For hybrid 128 in the panel, it was not described before in the text or figure as one of the 8 HR inducing hybrids, is it another hybrid or one of the controls?

Fig 2 - HR for G94N+DPS is not convincing in the plant image. Here and elsewhere - include a description of white/yellow circles.

Fig 4A: Hybrid 04 carries Pip1 part 3 sequence, but it seems to have relatively similar HR intensity (especially index 5 - 7) as 01, 02, who carry N194. This does not fully support the claim in Line 219-220. Hybrid 04 induces HR in this figure but not in Figure 3.

Fig 5A: note in the legend that residues highlighted in red represent swapped residues.

Fig 6: correct repeated (E) in the legend.

Please also note the following:

-The Plant Cell now requires authors to complete and submit an author revisions checklist upon submission of a revised manuscript. The aim of the checklist is to aid authors in preparing a high-quality manuscript, facilitate the review and assessment of revised manuscripts, and help to ensure that journal standards are maintained across the board. If your manuscript is accepted, the completed checklist will be published as supplemental material attached to the article online. Please download a copy of the checklist (pdf fillable form) at this link, for submission with your revised manuscript: https://tpc.msubmit.net/html/Author_Revisions_Checklist.pdf.

-Supplemental materials should be restricted to large datasets and tables, presentation of replicates, and validation of reagents, methods, or genotypes. Any data that are used to support the major claims must be in the main manuscript. Supplemental figure legends must indicate what figure in the main manuscript is supported by the supplemental data presented. Please justify how each of the supplemental figures meet the criteria.

-Sampling methods and the nature of "biological replicates" should be described precisely (i.e. different plants, parts of plants, pooled tissue, independent pools of tissue, sampled at different times, etc.), along with a clear description of and rationale for any statistical analyses conducted. The reader should know exactly what was sampled; what forms the basis of the calculation of any means and statistical parameters reported. This is also necessary to ensure that proper statistical analysis was conducted.

We would like to thank the editor and three anonymous reviewers for making their critical comments, which we have used to improve the manuscript.

The editor:

Dear Dr. van der Hoorn:

We have received reviews of your manuscript entitled "Bioengineering secreted proteases converts divergent Rcr3 orthologs and paralogs into extracellular immune co-receptors." On the basis of the advice received, the board of reviewing editors would like to accept your manuscript for publication in The Plant Cell. This acceptance is contingent on revision based on the comments of our reviewers. In particular, please consider the following:

1) Both reviewers noted issues with how HR scores are represented throughout this manuscript. We concur that the present graphs are difficult to interpret and should be modified. In particular, it is not clear whether each construct is scored with the same number of leaves. If not, the use of bubble sizes to indicate number of injections with each score may be misleading. It is also unclear why some dots are inside the bubbles and some dots are outside the bubbles. Perhaps it would be easier to interpret with a box and whisker style graph? At a minimum, please add a value for the mean HR score and the total number of leaves injected for each construct (if the number varies from construct to construct). RESPONSE: We have added the mean HR score and the number of replicates to all HR tables. We have also separated the individual datapoints (which were randomly distributed and sometimes overlapped) and made the bubbles lighter grey so the datapoints are easier to count. The replicate numbers are very comparable within each graph. We trust that this presentation increases the transparency of the assay. The box and whisker style graph is an alternative presentation that may confuse the transparency of this assay.

2) The primary novelty of this work is the potential to engineer new disease resistance traits using an apoplastic decoy. However, this particular example seems fairly limited to detection of protease inhibitors. Please expand your discussion of the potential applications of this specific system and its limitations (e.g., can Rcr3-Cf2 be engineered to recognize proteins secreted by other fungal species?). RESPONSE: The Rcr3/Cf-2 perception system is restricted to pathogen-secreted PLCP inhibitors, but since most apoplastic pathogens will secrete these inhibitors to suppress the activity of PLCPs, which most plants secrete in the apoplast, it is conceivable that this perception system can be bioengineered to include many, if not most bacterial, fungal and oomycete pathogens that colonise the apoplast. So the potential is huge. We have explained this potential in the last paragraph of the discussion.

3) The protease inhibition assay employed in Figure 2 needs to be explained in the legend and in the text. RESPONSE: We have added this explanation in the text and legend for Fig2 but this assay was also already explained in the text for Fig1, when this assay was first used.

4) Both reviewers have provided detailed suggestions for improving clarity of the writing. Please take these into consideration as you revise your manuscript. RESPONSE: We have revised the text following the suggestions of the two reviewers, as detailed below.

Reviewer #1 (Comments for the Author):

The manuscript by Kourelis and colleagues titled "Bioengineering secreted proteases converts divergent Rcr3 orthologs and paralogs into extracellular immune co-receptors" tackles engineering proteases involved in plant immunity. The authors use knowledge gained from Proteins in one species /cultivar to swap amino acid residues and engineer increased recognition and resistance in the form of a hypersensitive response. This type of incremental work and tinkering are essential in transferring information gathered from one study and applying it to other proteins, which would help the community in identifying common mechanistic insights. I applaud the authors on the amount of work put in to generate and test these variants. The authors prove that by replacing specific amino acid

residues proteins, in this case Rcr3 and Pip1 can be engineered in different species, which was one of the primary goals of the study.

I am also not convinced with the claim in the abstract that this study advances our understanding of the evolution of Avr2 perception. I agree that the engineering part of the manuscript is solid and the authors should stick to insights gained from this rather than make claims about evolution that dont stand. RESPONSE: We have removed 'the evolution of' from the abstract.

The manuscript is largely well put together, but could do with some editing for clarity, especially given that the manuscript switches between different proteins in different species. It becomes difficult to follow the nomenclature and all the different amino acid variants. Please disregard my queries asking for the basis of classifying the Rcr3 protein into different parts, I see that its mentioned in the methods section that these are arbitrary. But this in a sense underscores my point about manuscript clarity. The figures supplied would also benefit from better illustration and organization. I specify points below aimed at improving the manuscript in its current form. RESPONSE: we have addressed these points with the answers below.

Major points:
1. Please rephrase "A single residue substitution in eggplant SmRcr3 is required for Avr2/Cf-2-dependent HR" to "A single residue substitution in eggplant SmRcr3 is sufficient for Avr2/Cf-2-dependent HR" RESPONSE: We have made this revision.

2. It is not clear in the manuscript the classification based on which the Rcr3 protein was split into parts. This information would be helpful in understanding why replacing certain parts lead to cf-2 signalling. RESPONSE: We have added 'arbitrary, similar-sized parts' to the first paragraphs of the results to explain this. We made a similar statement when explaining Rcr3/Pip1 hybrids.

3. Figure. 1A - could you provide a better quality figure and if possible consider a different colour scheme, so that the figure is easier to read. RESPONSE: We have replaced yellow by purple and uploaded a 600dpi TIF file so Fig1A is easier to read now.

4. Figure. 1C - the HR indexes are difficult to read. This is true for all figures with HR indices. RESPONSE: We have revised all HR indexes so that the figure is easier to interpret and datapoints can be counted.

5. Line 121 - "Most other variant residues in part-4 are also different between Rcr3 homologs that can trigger Cf-2/Avr2-dependent HR." I am confused with this point, arent the variants based on the difference between HR-triggering Rcr3 vs SmRcr3? RESPONSE: Yes they are, but we point out that these positions are also variant when comparing Rcr3 homologs within Solanum species, which all trigger HR (Kourelis et al., *Nat. Comm.* 2020). We have inserted '*Solanum'* to clarify this.

6. Lines 72-76 in the introduction suggest that the protease activity of Rcr3 is not required for Cf-2-dependent HR, so I find line 131 a bit misleading in claiming that the D244P substitution turns SmRcr3 into a protease that triggers Cf-2-dependent HR. Perhaps, it is more accurate to state that SmRcr3 with the D244P substitution is capable of triggering Cf-2-dependent HR? Please clarify and elaborate in the text, is the above is incorrect. RESPONSE: We have replaced 'protease' by 'protein'.

7. The image presented in Fig. 1D and the HR index suggest that the D244P substitution while displaying HR in SmRcr3 is not comparable to Rcr3. Am I correct in interpreting this? And could the authors elaborate on this either way? RESPONSE: Does the reviewer mean that SmRcr3(D244P) is still less active than Rcr3? Yes, it seems, though the fact that we can trigger HR with a protease that is so different from Rcr3 is truly remarkable. We have added this observation to the main text.

8. Line 138 - I am not sure 'resurrected' is the best word to describe Rcr3a, but this is a semantic choice and I leave it to the authors to change or retain this. I would strongly suggest that they find a more suitable word. RESPONSE: We used the same terminology in the Nat. Comm. Manuscript so we prefer to be consistent. It is stated in quotes anyhow.

9. Suggestion - G194N instead of N194 in the title (line 134) would be more informative. RESPONSE: We have made this revision.

10. The basis of Fig. 2 is mainly dependent on "DPS" insertion and a Asparagine (N) at position #194. Substitution of the above residues in NtRcr3 is clear (Fig. 2c), however I am not convinced with the data presented for "rNbRcr3" (Fig.2b). In the absence of deletion of these three residues in Rcr3, it is difficult to interpret the importance of these residues. That said, I think that deletion of these residues in Rcr3 will be tangential to his work. So I would ask the authors to provide a better image and if it helps add a supplementary data figure with just three comparisons Rcr3 vs rNbRcr3 vs G194N+DPS. RESPONSE: A better image of the HR induced by r*Nb*Rcr3G194N+DPS has been added as ***NEW*** Supplemental **Figure S1**.

11. Line 166 - It is not clear what D329H refers to? Is it D329 in SlPip1 which naturally occurs at H329 in SpPip1? I strongly encourage the authors to go through all such attributions, and ensure that these are easier to read. RESPONSE: We have rephrased this sentence to clarify.

12. See point 2, what was the basis of splitting Rcr3 into seven "distinct" fragments? RESPONSE: We have replaced 'distinct fragments' by 'arbitrary similarly-sized fragments'.

13. Lines 292-293 need to be qualified as a possibility, as they are rightly done in the preceding lines. RESPONSE: We have replaced 'can' by 'might' as this is a hypothesis indeed.

14. Considering that Fig. 6c is based on Alphafold2, have the Authors explored the possibility of alternate structural arrangements of M338 and I285? Would a different arrangement open the substrate-binding pocket to accommodate other substrates? RESPONSE: We have analysed the five models predicted by AlphaFold2 for Pip1 and Rcr3 and found that the size of the S2 pockets was very consistent. Measurements of the distance between the active site Cys and the two residues are consistently smaller for Rcr3 than for Pip1:

	**Pip1**	**Rcr3**	**Pip1**	**Rcr3**
	C155-V338	C155-M338	C155-L285	C155-I285
Model-1	11.2	9.4	9.5	8.1
Model-2	10.8	9.5	9.4	8.2
Model-3	10.9	9.2	9.5	8.2
Model-4	10.4	9.3	9.5	8.1
Model-5	11.2	9.5	9.5	8.3

15. Line 298-299 - "Thus, the larger S2 pocket in Pip1 explains why it can accommodate substrates with P2=Phe, in contrast to Rcr3, which prefers smaller residues at P2." - This conclusion is based on a premise built over an unverified model. The authors would be better off toning down these claims. RESPONSE: We have rephrased this into: Thus, the putative larger S2 pocket in Pip1 might be why it can accommodate substrates with P2=Phe, …'

Minor points:
1. Specify tomato Rcr3 in line 37. I understand that this is a continuation of the previous line where Rcr3 in tomato was mentioned, however, I had to re-read this line a couple of times and think that simply assigning it as SlRcr3 or something equivalent for the introgressed line would make it easier to read. RESPONSE: We have revised this sentence into 'crucial residues from tomato Rcr3', as requested.

2. Line 72 - Please expand on "not processed by Rcr3", I am assuming this refers to Rcr3's protease activity. RESPONSE: We have changed this into 'proteolytically processed' to clarify this.

3. Line 73-76 - please consider splitting this into two sentences. RESPONSE: We have changed this into two sentences.

4. Line 178 - cause → caused RESPONSE: We have made this revision.

5. Legend of Fig. 5a is incomplete RESPONSE: We have completed this legend.

6. Line 287 - "also" is misplaced. RESPONSE: We have moved 'also' within this sentence.

7. Line 331 – misspelled RESPONSE: Sorry, we could not identify this error.

8. Line 398 - please rephrase in the first line of the sentence, interaction between Cf-2 and what? I assume authors mean Avr2 and Rcr3, which come at the very end of the sentence. RESPONSE: We have rephrased this into: 'demonstrate interactions of Avr2/Rcr3 or Rcr3 with Cf-2'.

9. Line 409 - "Nevertheless" can be deleted RESPONSE: We have deleted this word.

Reviewer #2 (Comments for the Author):

The manuscript describes the bioengineering of the plant-secreted immune proteases Rcr3 and Pip1 for gain of function in Cf-2 mediated recognition. The manuscript is well-written and should be of interest to the scientific community given the recent intense interest in receptor engineering. However, the findings would be of more interest by combining some selection analyses including newly available genomes.

Major comments:
The authors discussed different selection pressures may be present in a region and lineage specific bases. Specifically, HR-related Rcr3 residues may be under purifying or neutral selection. The paper would be strengthened by directly testing this, potentially with codon-substitution models from CodeML. RESPONSE: This is related to the following lines in the discussion "*This should cause opposing selection forces in two distinct areas of Rcr3: residues required for Cf-2-dependent HR will be under selection to increase interactions with Cf-2 only in Solanum and evolve under neutrality in other solanaceous plants*". While we agree that investigating the selection pressures on HR-related Rcr3 residues using codon-substitution models from CodeML would be interesting, some of this work has already been conducted for Rcr3 in *S. peruvianum* (Hoerger et al., 2012). Additionally, we previously showed that the locus that gave rise to Cf-2 is only found in *Solanum* species, and is rapidly diversifying (Kourelis et al., 2020). However, we do not know exactly when Cf-2 itself evolved and in which species it can be found. This therefore complicates the above analysis. Furthermore, in potato and some tomato species Rcr3 has been duplicated, which could result in differential pressures on different Rcr3 paralogs (Ilyas et al., 2015; Hoerger et al., 2012). We have toned down the claim in the subsequent sentence, replacing the word "probably" with "could".

When analyzing Rcr3 sequences in eggplant and progenitor species - are there any variants with D224P? RESPONSE: We examined multiple *Solanum melongena* genomes as well as the related species *Solanum macrocarpon*. Our analysis shows that in the *S. melongena*-HQ eggplant genome (Smechr0201177.1https://doi.org/10.1038/s41438-020-00391-0), the *S. melongena* 67/3 genome (SMEL4_02g020910.1.01 in version 4.0 or SMEL4.1_02g010450.1.0 in version 4.1; https://doi.org/10.1111/tpj.15313, the S. melongena Guiqie1 genome (SolGenomics), and the *S. melongena* PI 200854 and PI 180485, as well as the *S. macrocarpon* PI 441915 01 genome, the D244 position is conserved as aspartic acid (D) and not proline (P). Thus, our findings confirm that the D244P variant does not naturally occur in the genomes we analyzed, and D244 is conserved across these eggplant varieties and related species.

HR index: An average HR index should also be included in the figures, which will allow for a more straightforward comparison of HR intensity between constructs. Fig 3D: I suggest highlighting the HR inducing hybrid names with underscore or color for a more consistent and easier visual interpretation. For the raw dots in the bubble plot, it seems that not all hybrids have the 12 datapoints (are these replicates described in the legend?) plotted in the bubbles (E.g. hybrid 28, 32, 44, 48, 60 only have 7, 7, 6, 5, 7 data points, respectively). The uneven number of plotted data points may affect the size of bubbles as the bubbles seems to be direct representation of number of plotted data points. RESPONSE: We have added the mean HR score and the replicate number, which are comparable within each subfigure. The datapoints were partly overlapping and have been separated so they can be

counted. The background is also lighter so see the datapoints better. Underlining and/or colouring the names would be confusing since we have several proteases with intermediate phenotypes. We have, however, printed the mean numbers larger and in grey scale to stress HR responses.

AlphaFold Modeling: Please put Fig 7 data earlier to highlight in each fig for GOF regions that are required for recognition. Does a structure-based alignment of the native Rcr3 homologs including Pip1, Rcr3 from various species and bioengineered Rcr3 variants in this study reveal that HR-inducing homologs structures form a distinct cluster from non-HR inducing homologs? Provide pTM scores as quality control for the AlphaFold models in the supplemental material. RESPONSE: We have moved the structural models to the respective sections, as suggested, and mentioned the pTM values in the main text (all pTM=0.94). We appreciate the suggestion to use structure-based alignment alongside AlphaFold modeling. However, based on our assessment, sequence clustering already provides comprehensive insights into the conservation and variation among Rcr3 homologs, including bioengineered variants. Given that structural modeling would not add substantial new information to our sequence-based findings, we have chosen not to include it in this study.

Protein expression: It would be good to demonstrate for a few key GOF mutants and negative controls that the PLCP is expressed by western blot. RESPONSE: We cannot do this because the proteases are not epitope tagged and we do not know which part of the proteins are recognised by our polyclonal antibodies. Consequently, the western bots of the Rcr3/Pip1 hybrids would not be trustworthy. By contrast, we used activity-based labelling, which is a far more consistent, reliable measure of: 1) that the protease is active because it would not be labelled otherwise; 2) it is a mature protease, based on its MW in the gel; 3) the relative abundance, assuming that we have saturated labelling during the used long incubation times.

Other comments:
Fig 3C: It seems that this fluorescence panel does not contain native Pip and Rcr3 as controls, and hybrid 48, 32, 128 seems to have very weak change of Fluorescence. For hybrid 128 in the panel, it was not described before in the text or figure as one of the 8 HR inducing hybrids, is it another hybrid or one of the controls? RESPONSE: Although some of the signals are weak, they differ slightly in size and intensity, consistent with being variants, so this signal is not a caused by an endogenous protease, and all these signals are reduced or absent upon preincubation with Avr2, which supports our conclusion that they are sensitive to Avr2 inhibition. Construct 128 encodes Rcr3. We have renamed this in Figure 3C.

Fig 2 - HR for G94N+DPS is not convincing in the plant image. Here and elsewhere - include a description of white/yellow circles. RESPONSE: We have added a description of the white/yellow circles to the legend, and we added a ***NEW*** Supplemental **Figure S1** showing HR induced by r*Nb*Rcr3(G194N+DPS).

Fig 4A: Hybrid 04 carries Pip1 part 3 sequence, but it seems to have relatively similar HR intensity (especially index 5 - 7) as 01, 02, who carry N194. This does not fully support the claim in Line 219-220. Hybrid 04 induces HR in this figure but not in Figure 3. RESPONSE: Part-3 has a strong quantitative contribution to the HR, and the observed differences are dependent on when the HR is imaged/scored. At earlier time points (4dpi) the HR is not as pronounced and there is a clear quantitative difference, whereas at later time points (7dpi onwards) the difference is less distinct. We have now further clarified this in the text describing Figure 3D and Figure 4A.

Fig 5A: note in the legend that residues highlighted in red represent swapped residues. RESPONSE: We have completed this legend to clarify this.

Fig 6: correct repeated (E) in the legend. RESPONSE: We have corrected this typographic error.

Please also note the following:

-The Plant Cell now requires authors to complete and submit an author revisions checklist upon

submission of a revised manuscript. The aim of the checklist is to aid authors in preparing a high-quality manuscript, facilitate the review and assessment of revised manuscripts, and help to ensure that journal standards are maintained across the board. If your manuscript is accepted, the completed checklist will be published as supplemental material attached to the article online. Please download a copy of the checklist (pdf fillable form) at this link, for submission with your revised manuscript: https://tpc.msubmit.net/html/Author_Revisions_Checklist.pdf. RESPONSE: This checklist has been completed and uploaded with the revised manuscript.

-Supplemental materials should be restricted to large datasets and tables, presentation of replicates, and validation of reagents, methods, or genotypes. Any data that are used to support the major claims must be in the main manuscript. Supplemental figure legends must indicate what figure in the main manuscript is supported by the supplemental data presented. Please justify how each of the supplemental figures meet the criteria. RESPONSE: Figure S1 is a replicate experiment showing HR; Figure S2 shows which Rcr3/Pip1 hybrids are active proteases, which is summarised in Figure 3B. Figure S3 shows which Rcr3/Pip1 hybrid is Avr2 sensitive, summarised in Figure 3B and 3C. Figure S4 shows the classic HR assays in tomato, triggered by Rcr3/Avr2 injection, which have been replaced by much less tedious agroinfiltration assays in the manuscript, with the same conclusions. Figure S5 summarises the reasoning towards specific residues, as explained in the main text.

-Sampling methods and the nature of "biological replicates" should be described precisely (i.e. different plants, parts of plants, pooled tissue, independent pools of tissue, sampled at different times, etc.), along with a clear description of and rationale for any statistical analyses conducted. The reader should know exactly what was sampled; what forms the basis of the calculation of any means and statistical parameters reported. This is also necessary to ensure that proper statistical analysis was conducted. RESPONSE: We state the following in the material and methods: “Replicate numbers given indicate independent infiltrations of different leaves from multiple plants. For quantitation, HR cell death phenotypes were scored in a range from 0 (no visible necrosis) to 7 (fully confluent necrosis) as in Kourelis et al., 2022 and plotted using a R script modified from Bentham et al., 2023. Statistical analysis was conducted using the besthr R package (MacLean, 2020).”

Dear Dr. Renier van der Hoorn:

We are pleased to inform you that your paper entitled "Bioengineering secreted proteases converts divergent Rcr3 orthologs and paralogs into extracellular immune co-receptors" has been accepted for publication in The Plant Cell, pending a final editorial review by a science editor. At this stage, your manuscript will be evaluated by a science editor with respect to its presentation of scientific content, compliance with journal policies, and presentation for a broad readership. The Plant Cell has contracted with Plant Editors (planteditors.com) to provide this service to our authors, and you will soon receive additional information on this process.

Please note that each author needs to link their ORCID identifier to their account in the system before your manuscript can be published. If any authors do not have an ORCID linked to their account, they will receive a message with a link to complete this task. Please ensure that ALL of your coauthors have completed this task as soon as possible.

ASPB offers an OPEN option that allows authors to have their online articles available for free to all users immediately upon publication. For more information about the ASPB OPEN option, refer to the Final Submission Checklist Form.

The Plant Cell and The Arabidopsis Information Resource (TAIR) are collaborating to collect functional annotation data about Arabidopsis genes from authors. This includes information about the gene's molecular function (e.g., kinase activity, ATP synthetase activity), the biological process/es it is involved in (e.g., endosperm development, threonine biosynthesis), its subcellular location (e.g., nucleus, ER), anatomical or developmental expression pattern (e.g., leaf, ovule, flower stage 10, seedling stage), or its partner in a protein-protein interaction (e.g., AT1G01010 interacts with AT1G01020).

If your paper contains results falling into one or more of these categories for Arabidopsis genes, we request that you now submit these data for inclusion in TAIR by filling in the form provided at the following URL: https://www.arabidopsis.org/doc/submit/functional_annotation/123. If you need further clarification on what types of data can be submitted please contact curator@arabidopsis.org.

Finally, we encourage your submission of artwork for the journal cover. Monthly issues will have an online cover image and selected covers will be used for posters, other promotional items, and "wallpaper" for mobile devices. For more information, refer to Cover Submission in the Instructions for Authors https://tpc.msubmit.net/cgi-bin/main.plex?form_type=display_auth_instructions.

We look forward to seeing your paper published.

Sincerely,

The Plant Cell Editorial Board

(Please note that due to a potential conflict of interest, EIC Blake Meyers did not participate in evaluating this manuscript).

=====
IMPORTANT REMINDER: PEER REVIEW REPORTS
=====

If you opted to publish a peer review report along with your article during the original submission process, it will be prepared by the editorial staff and publicly posted with your manuscript, inside the zip file that contains any other supplemental material. As a reminder, the peer review report is a public record of all comments from editors and reviewers, as well as your prior responses, as you received them in the decision letters for each draft of your manuscript. If you agreed to publish this report and have changed your mind, or are not sure if you selected this option, please contact the editorial office as soon as possible before signing the license agreement from our publisher.

=====
REVIEWER COMMENTS
=====

----FOR ASPB OFFICE USE ONLY (DO NOT EDIT)----
MSID: 38393
Corresponding Author: Prof. van der Hoorn
