# Supplementary material for: Bioengineering secreted proteases converts divergent Rcr3 orthologs and paralogs into extracellular immune co-receptors
Source: Plant Cell. 2024 Jun 26;36(9):3260–76. doi: 10.1093/plcell/koae183 (PMC11371160; doi:10.1093/plcell/koae183)
Supplement: koae183_Supplementary_Data [file koae183_supplementary_data.zip › Supplementary Dataset S3.zip!/S3_Dataset/BestHR_Hybrids.pdf]

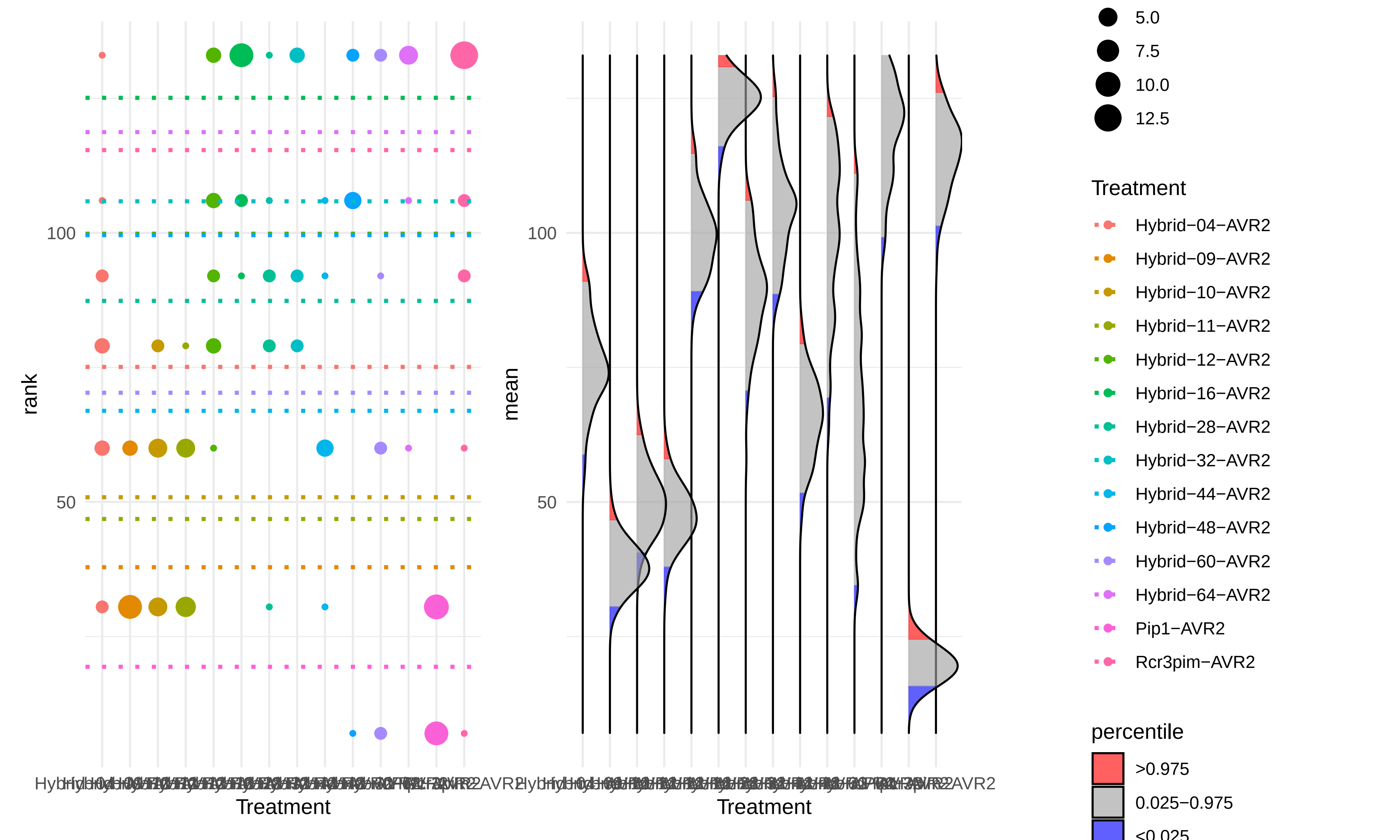

rank
100
50
Treatment
mean
100
50
Treatment
5.0
7.5
10.0
12.5
Treatment
Hybrid−04−AVR2
Hybrid−09−AVR2
Hybrid−10−AVR2
Hybrid−11−AVR2
Hybrid−12−AVR2
Hybrid−16−AVR2
Hybrid−28−AVR2
Hybrid−32−AVR2
Hybrid−44−AVR2
Hybrid−48−AVR2
Hybrid−60−AVR2
Hybrid−64−AVR2
Pip1−AVR2
Rcr3pim−AVR2
percentile
>0.975
0.025−0.975
<0.025
