# Supplementary material for: Bioengineering secreted proteases converts divergent Rcr3 orthologs and paralogs into extracellular immune co-receptors
Source: Plant Cell. 2024 Jun 26;36(9):3260–76. doi: 10.1093/plcell/koae183 (PMC11371160; doi:10.1093/plcell/koae183)
Supplement: koae183_Supplementary_Data [file koae183_supplementary_data.zip › Supplementary Dataset S3.zip!/S3_Dataset/BestHR_NbRCR3.pdf]

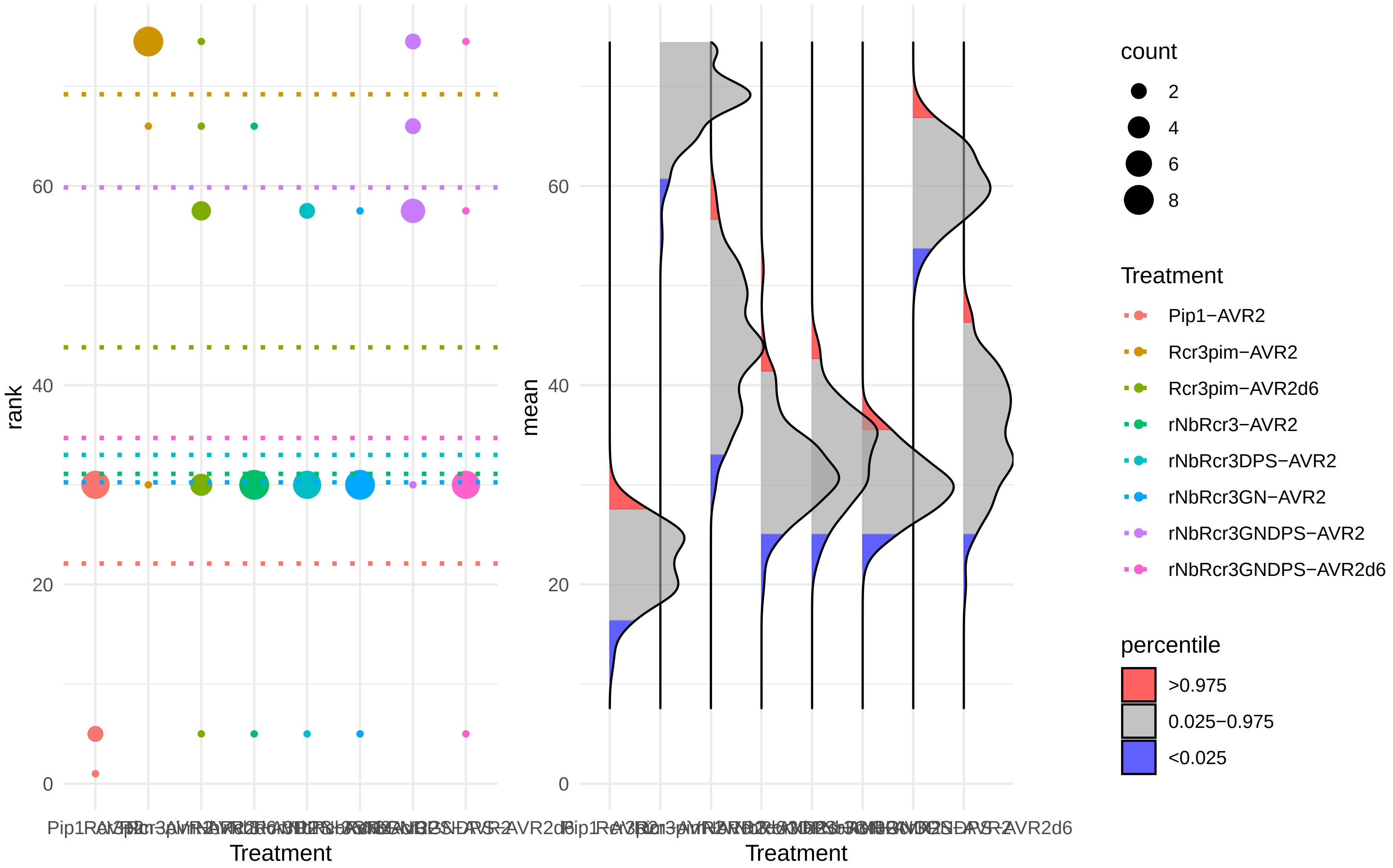

rank
mean
Treatment
Treatment
count
2
4
6
8
Treatment
Pip1−AVR2
Rcr3pim−AVR2
Rcr3pim−AVR2d6
rNbRcr3−AVR2
rNbRcr3DPS−AVR2
rNbRcr3GN−AVR2
rNbRcr3GNDPS−AVR2
rNbRcr3GNDPS−AVR2d6
percentile
>0.975
0.025−0.975
<0.025
