# Supplementary material for: Bioengineering secreted proteases converts divergent Rcr3 orthologs and paralogs into extracellular immune co-receptors
Source: Plant Cell. 2024 Jun 26;36(9):3260–76. doi: 10.1093/plcell/koae183 (PMC11371160; doi:10.1093/plcell/koae183)
Supplement: koae183_Supplementary_Data [file koae183_supplementary_data.zip › Supplementary Dataset S3.zip!/S3_Dataset/BestHR_pJK235_03.pdf]

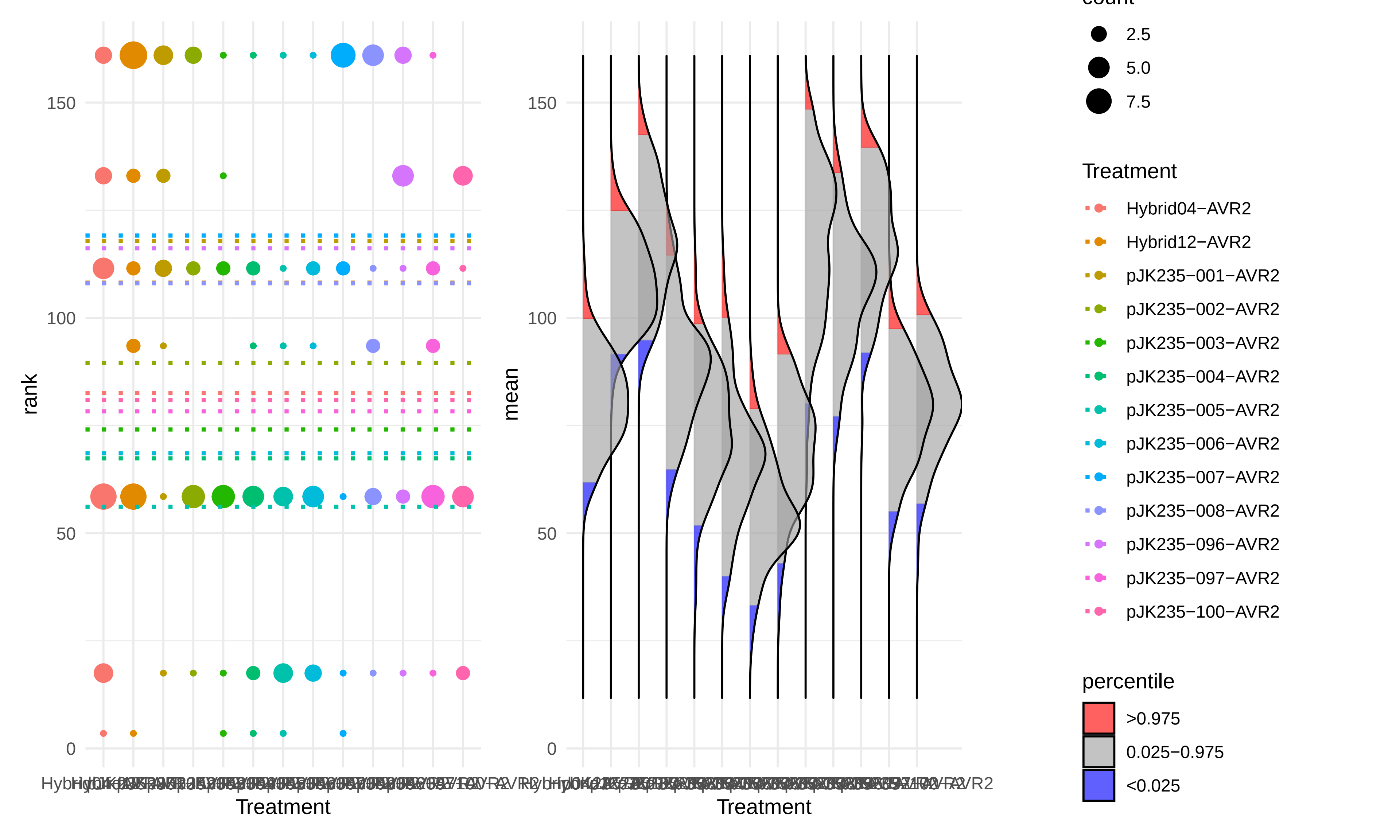

count
2.5
5.0
7.5
Treatment
Hybrid04−AVR2
Hybrid12−AVR2
pJK235−001−AVR2
pJK235−002−AVR2
pJK235−003−AVR2
pJK235−004−AVR2
pJK235−005−AVR2
pJK235−006−AVR2
pJK235−007−AVR2
pJK235−008−AVR2
pJK235−096−AVR2
pJK235−097−AVR2
pJK235−100−AVR2
percentile
>0.975
0.025−0.975
<0.025
rank
mean
150
100
50
0
Treatment
Treatment
