# Supplementary material for: Bioengineering secreted proteases converts divergent Rcr3 orthologs and paralogs into extracellular immune co-receptors
Source: Plant Cell. 2024 Jun 26;36(9):3260–76. doi: 10.1093/plcell/koae183 (PMC11371160; doi:10.1093/plcell/koae183)
Supplement: koae183_Supplementary_Data [file koae183_supplementary_data.zip › Supplementary Dataset S3.zip!/S3_Dataset/BestHR_pJK235_05.pdf]

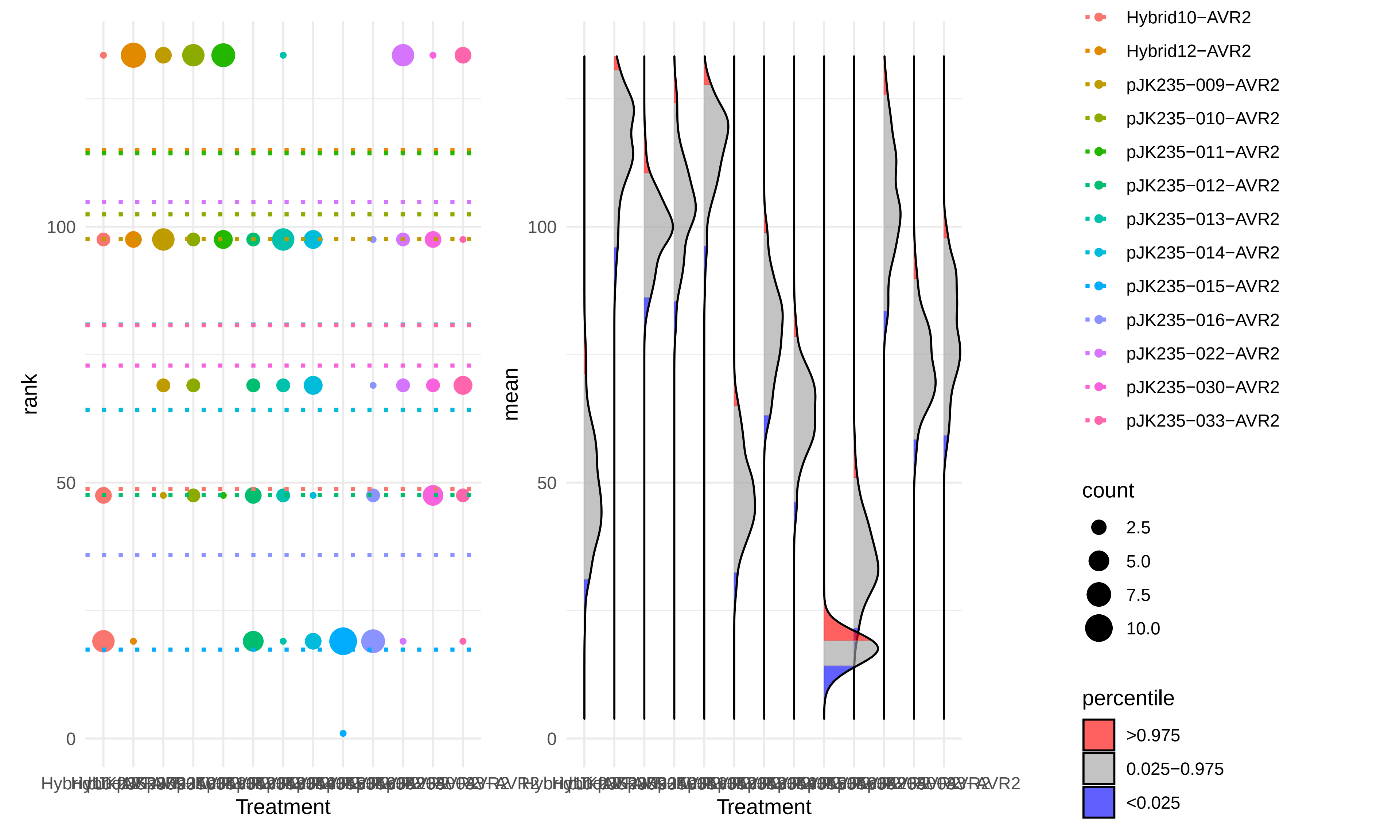

rank
100
50
0
Treatment
mean
100
50
0
Treatment
Hybrid10−AVR2
Hybrid12−AVR2
pJK235−009−AVR2
pJK235−010−AVR2
pJK235−011−AVR2
pJK235−012−AVR2
pJK235−013−AVR2
pJK235−014−AVR2
pJK235−015−AVR2
pJK235−016−AVR2
pJK235−022−AVR2
pJK235−030−AVR2
pJK235−033−AVR2
count
2.5
5.0
7.5
10.0
percentile
>0.975
0.025−0.975
<0.025
