# Supplementary material for: Bioengineering secreted proteases converts divergent Rcr3 orthologs and paralogs into extracellular immune co-receptors
Source: Plant Cell. 2024 Jun 26;36(9):3260–76. doi: 10.1093/plcell/koae183 (PMC11371160; doi:10.1093/plcell/koae183)
Supplement: koae183_Supplementary_Data [file koae183_supplementary_data.zip › Supplementary Dataset S3.zip!/S3_Dataset/BestHR_pJK235_06.pdf]

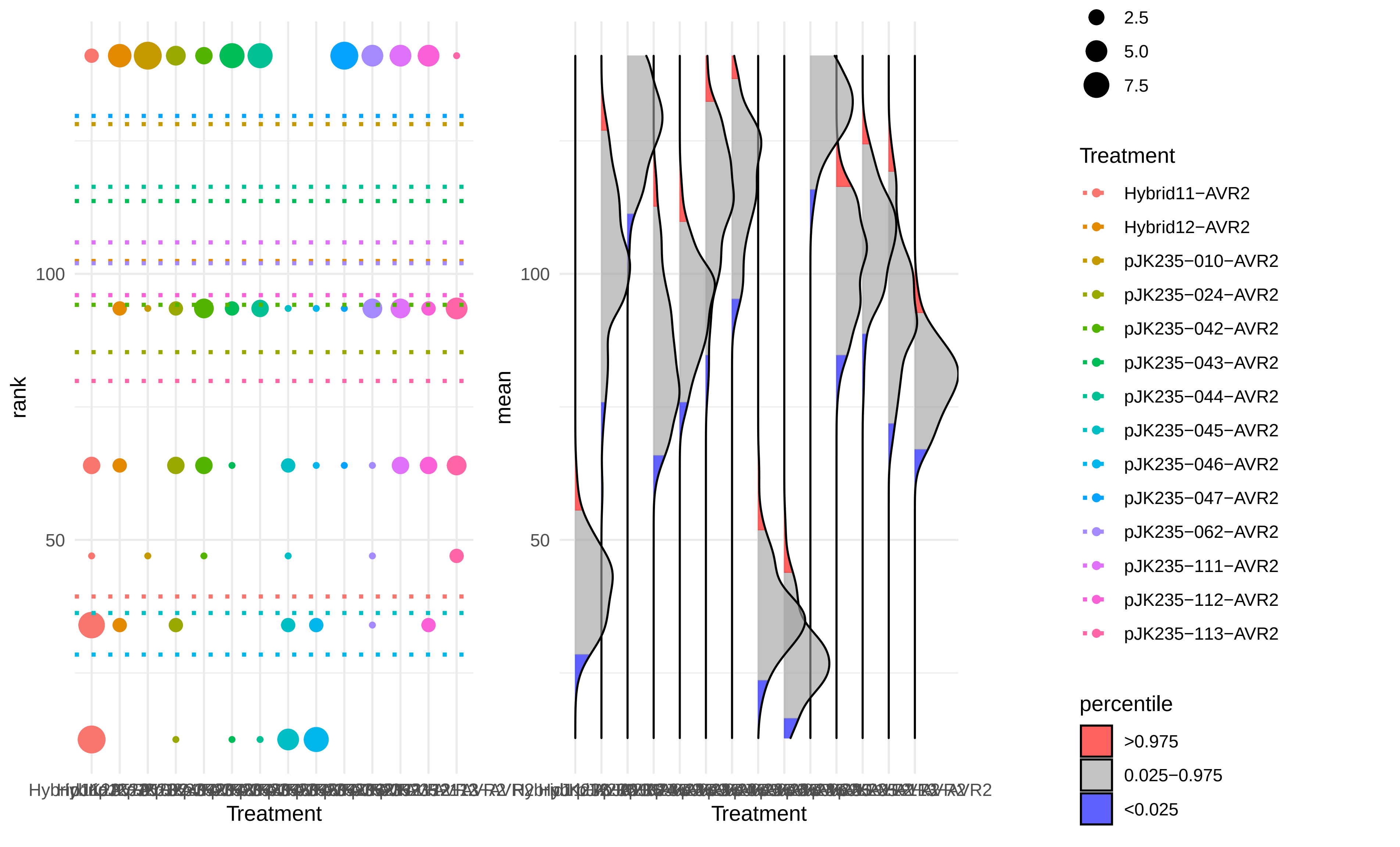

rank
100
50
Treatment
mean
100
50
Treatment
2.5
5.0
7.5
Treatment
Hybrid11−AVR2
Hybrid12−AVR2
pJK235−010−AVR2
pJK235−024−AVR2
pJK235−042−AVR2
pJK235−043−AVR2
pJK235−044−AVR2
pJK235−045−AVR2
pJK235−046−AVR2
pJK235−047−AVR2
pJK235−062−AVR2
pJK235−111−AVR2
pJK235−112−AVR2
pJK235−113−AVR2
percentile
>0.975
0.025−0.975
<0.025
