# Supplementary material for: Bioengineering secreted proteases converts divergent Rcr3 orthologs and paralogs into extracellular immune co-receptors
Source: Plant Cell. 2024 Jun 26;36(9):3260–76. doi: 10.1093/plcell/koae183 (PMC11371160; doi:10.1093/plcell/koae183)
Supplement: koae183_Supplementary_Data [file koae183_supplementary_data.zip › Supplementary Dataset S3.zip!/S3_Dataset/BestHR_SmRCR3.pdf]

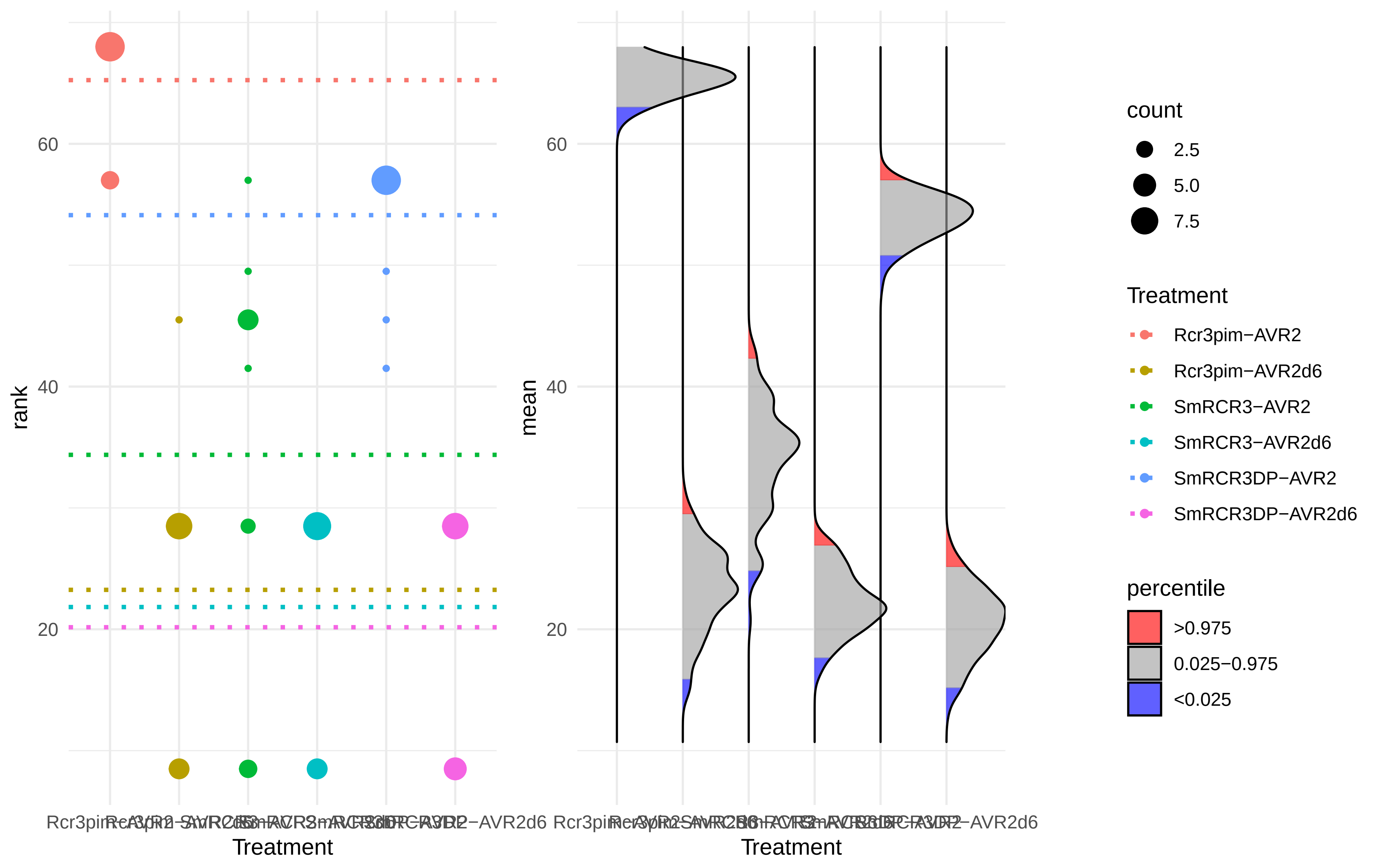

rank
60
40
20
Treatment
mean
60
40
20
Treatment
count
2.5
5.0
7.5
Treatment
Rcr3pim−AVR2
Rcr3pim−AVR2d6
SmRCR3−AVR2
SmRCR3−AVR2d6
SmRCR3DP−AVR2
SmRCR3DP−AVR2d6
percentile
>0.975
0.025−0.975
<0.025
