# Supplementary material for: Bioengineering secreted proteases converts divergent Rcr3 orthologs and paralogs into extracellular immune co-receptors
Source: Plant Cell. 2024 Jun 26;36(9):3260–76. doi: 10.1093/plcell/koae183 (PMC11371160; doi:10.1093/plcell/koae183)
Supplement: koae183_Supplementary_Data [file koae183_supplementary_data.zip › Supplementary Dataset S3.zip!/S3_Dataset/BestHR_SmRCR3_fragment.pdf]

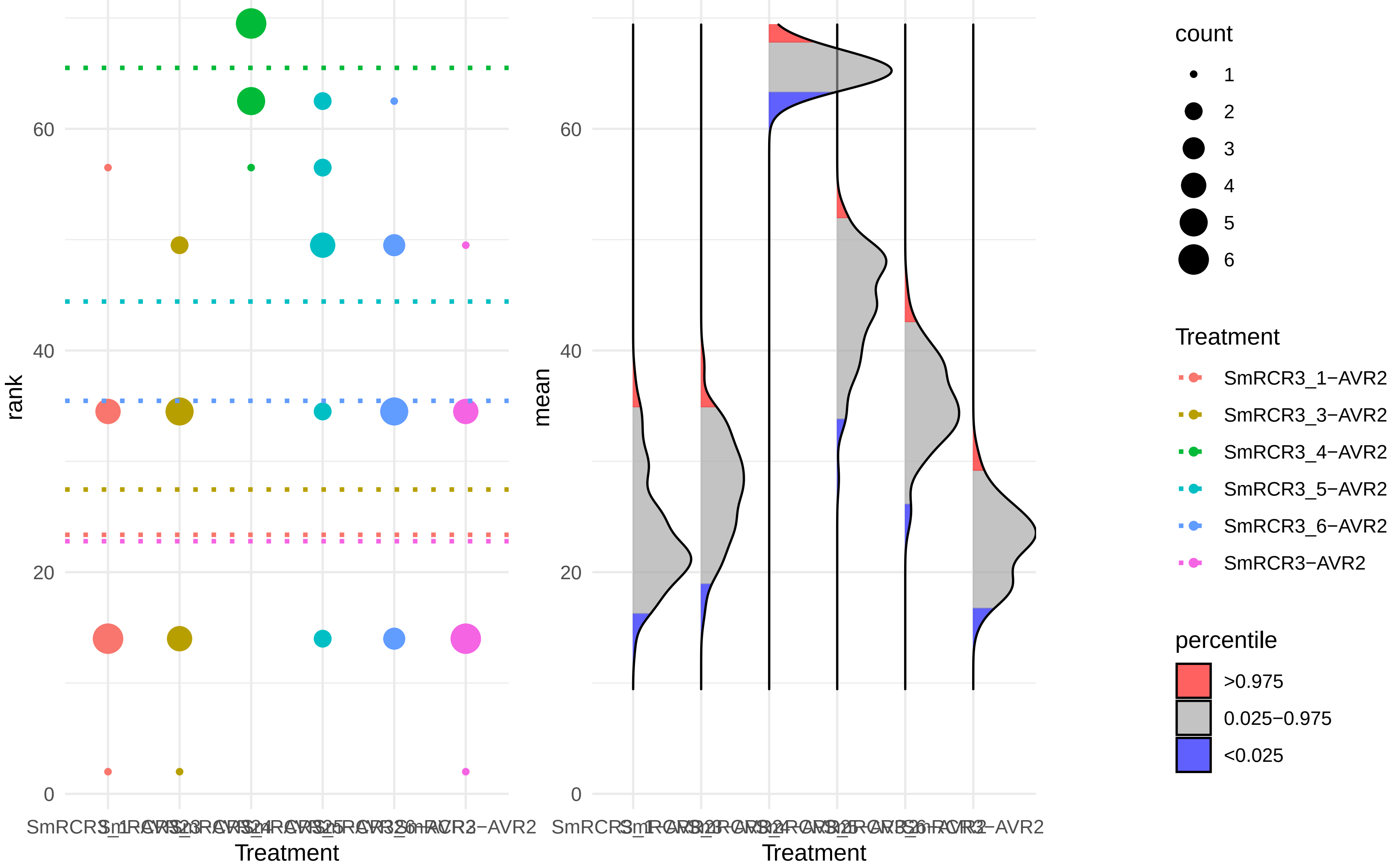

60
40
20
0
rank
Treatment
60
40
20
0
mean
Treatment
count
1
2
3
4
5
6
Treatment
SmRCR3_1−AVR2
SmRCR3_3−AVR2
SmRCR3_4−AVR2
SmRCR3_5−AVR2
SmRCR3_6−AVR2
SmRCR3−AVR2
percentile
>0.975
0.025−0.975
<0.025
